# Characterising the association of latency with α_1_-antitrypsin polymerisation using a novel monoclonal antibody

**DOI:** 10.1016/j.biocel.2014.11.005

**Published:** 2015-01

**Authors:** Lu Tan, Juan Perez, Marianna Mela, Elena Miranda, Keith A Burling, Farshid N Rouhani, Dawn L DeMeo, Imran Haq, James A Irving, Adriana Ordóñez, Jennifer A Dickens, Mark Brantly, Stefan J Marciniak, Graeme J M Alexander, Bibek Gooptu, David A Lomas

**Affiliations:** a Department of Medicine, University of Cambridge, Cambridge Institute for Medical Research, Cambridge, UK; b Eawag, Swiss Federal Institute of Aquatic Science and Technology, Dübendorf, Switzerland; c Department of Cell Biology, Genetics and Physiology, University of Málaga, Málaga, Spain; d Division of Gastroenterology & Hepatology, University Department of Medicine, Cambridge University Hospitals, Cambridge, UK; e Department of Biology and Biotechnologies Charles Darwin and Pasteur Institute-Cenci Bolognetti Foundation—University of Rome La Sapienza, Rome, Italy; f Core Biochemical Assay Laboratory, Cambridge University Hospitals NHS Foundation Trust, Cambridge, UK; g Department of Medicine, College of Medicine, University of Florida, Gainesville, FL, USA; h Channing Division of Network Medicine, Department of Medicine, Brigham and Women's Hospital, Boston, MA, USA; i Wolfson Institute for Biomedical Research, University College London, London, UK; j Division of Asthma, Allergy and Lung Biology, King's College London, Guy's Hospital, 5th Floor, Tower Wing, London, UK

**Keywords:** α1-Antitrypsin, Latency, Polymerisation, Monoclonal antibodies, Augmentation therapy

## Abstract

α_1_-Antitrypsin is primarily synthesised in the liver, circulates to the lung and protects pulmonary tissues from proteolytic damage. The Z mutant (Glu342Lys) undergoes inactivating conformational change and polymerises. Polymers are retained within the hepatocyte endoplasmic reticulum (ER) in homozygous (PiZZ) individuals, predisposing the individuals to hepatic cirrhosis and emphysema. Latency is an analogous process of inactivating, intra-molecular conformational change and may co-occur with polymerisation. However, the relationship between latency and polymerisation remained unexplored in the absence of a suitable probe. We have developed a novel monoclonal antibody specific for latent α_1_-antitrypsin and used it in combination with a polymer-specific antibody, to assess the association of both conformers *in vitro*, in disease and during augmentation therapy. *In vitro* kinetics analysis showed polymerisation dominated the pathway but latency could be promoted by stabilising monomeric α_1_-antitrypsin. Polymers were extensively produced in hepatocytes and a cell line expressing *Z* α_1_-antitrypsin but the latent protein was not detected despite manipulation of the secretory pathway. However, α_1_-antitrypsin augmentation therapy contains latent α_1_-antitrypsin, as did the plasma of 63/274 PiZZ individuals treated with augmentation therapy but 0/264 who were not receiving this medication (*p* < 10^−14^). We conclude that latent α_1_-antitrypsin is a by-product of the polymerisation pathway, that the intracellular folding environment is resistant to formation of the latent conformer but that augmentation therapy introduces latent α_1_-antitrypsin into the circulation. A suite of monoclonal antibodies and methodologies developed in this study can characterise α_1_-antitrypsin folding and conformational transitions, and screen methods to improve augmentation therapy.

## 1 Introduction

**Fig. 1:**
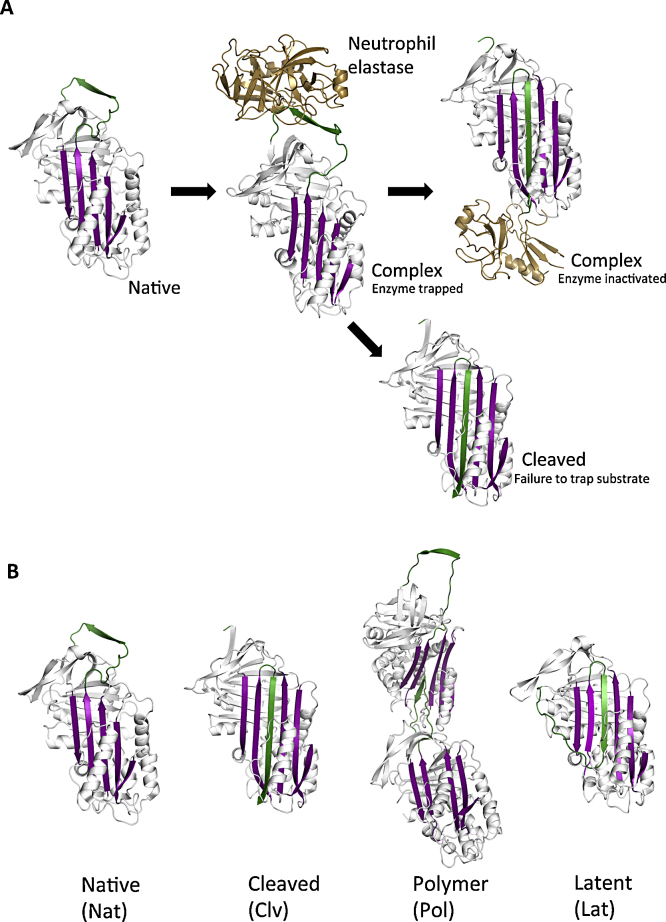
*Function of α_1_-antitrypsin and structures of its conformers.* (A) The structure of α_1_-antitrypsin (native) is based on three β-sheets (purple), nine α-helices and a flexible reactive centre loop (green) that is recognised as a pseudosubstrate by neutrophil elastase (Elliott et al., 1998) (complex). Upon binding, the reactive centre loop of α_1_-antitrypsin is cleaved and incorporated into β-sheet A. This translocates neutrophil elastase from the upper to the lower pole of the protein which distorts the catalytic site and inactivates the enzyme. Interaction of α_1_-antitrypsin with a non-target protease results in the cleaved conformer in which the reactive loop is cleaved and inserted into β-sheet A without the formation of an inhibitory complex (cleaved). (B) Major conformations of α_1_-antitrypsin include the native (Nat), cleaved (Clv), polymeric (Pol) and latent (Lat) forms. Different models for the structure of the polymer have been proposed (Carrell et al., 1994, Dafforn et al., 1999, Ekeowa et al., 2010, Gooptu et al., 2000, Haq et al., 2013, Lomas et al., 1992, McGowan et al., 2006, Sharp et al., 1999, Yamasaki et al., 2008, Yamasaki et al., 2011, Zhang et al., 2008) including the classical model, as shown (Pol), in which the reactive centre loop of one molecule is incorporated into the open β-sheet A of another molecule. The latent conformer (Lat) features the insertion of the reactive centre loop into the same molecule. The cleaved, polymeric and latent conformations have no inhibitory activity. (For interpretation of the references to color in this figure legend, the reader is referred to the web version of this article.)

α_1_-Antitrypsin is a member of the serine protease inhibitor (serpin) superfamily. It is predominantly synthesised in hepatocytes and circulates to the lung where it functions to protect the pulmonary tissue from proteolytic damage by inhibiting the enzyme neutrophil elastase (Fig. 1A). Individuals carrying two wild-type M alleles that encode functional α_1_-antitrypsin are denoted PiMM (Lomas et al., 1992, Perlmutter, 2004, Stoller and Aboussouan, 2012). The Z allele of α_1_-antitrypsin (Glu342Lys) causes 85-90% of the protein to misfold, undergo inactivating conformational change and either be degraded or form polymers that are retained within hepatocytes. The gain-of-function toxicity of polymeric α_1_-antitrypsin causes liver damage with nearly half of α_1_-antitrypsin deficient adults over the age of 50 having features of liver cirrhosis and occasionally hepatocellular carcinoma (Eriksson et al., 1986). The concomitant lack of circulating α_1_-antitrypsin predisposes PiZZ homozygotes to early onset emphysema.

Polymerisation of *Z* α_1_-antitrypsin is a central event in the pathogenesis of α_1_-antitrypsin deficiency. It arises through aberrant structural behaviour within the α_1_-antitrypsin molecule that subverts a process of conformational change that is essential for normal function (Fig. 1B). Polymeric α_1_-antitrypsin is found within the Periodic Acid Schiff (PAS) positive inclusion bodies in hepatocytes (Ekeowa et al., 2010, Eriksson et al., 1986, Miranda et al., 2010). Polymers of α_1_-antitrypsin are also present in the circulation of PiZZ individuals (Janciauskiene et al., 2002, Schmid et al., 2012, Tan et al., 2014) and have been detected in the lung, kidney and skin (Elliott et al., 1998, Gross et al., 2009, Lawless et al., 2004, Mahadeva et al., 2005, Morris et al., 2011, Paakko et al., 1996, Venembre et al., 1994). The structure of the polymers that form within hepatocytes has been the subject of much debate. Several models of polymerisation have been proposed including the classical reactive centre loop-β-sheet A linkage (Fig. 1B, Pol) first proposed in 1992 (Dafforn et al., 1999, Ekeowa et al., 2010, Gooptu et al., 2000, Haq et al., 2013, Lomas et al., 1992); linkage *via* β-sheet C (Carrell et al., 1994, Zhang et al., 2008); an extra, lateral strand of β-sheet A (s7A) (McGowan et al., 2006, Sharp et al., 1999); β-hairpin (Yamasaki et al., 2008) and C-terminal (Yamasaki et al., 2011) domain swaps. Studies using the polymer-specific 2C1 monoclonal antibody (MAb) have shown that there are multiple forms of polymer depending on the conditions used to generate the polymer *in vitro* (Ekeowa et al., 2010, Miranda et al., 2010).

Serpins can adopt the inactive but intact monomeric latent conformation in which the reactive centre loop is inserted into the underlying β-sheet A in the absence of proteolytic cleavage (Fig. 1B, Lat). The principle models of serpin polymerisation relate formation of the latent conformer to polymerisation in different ways. In the “classical” (reactive centre loop-β-sheet A insertion) model of serpin polymerisation, the latent conformer is an alternative product of the pathway that leads to polymerisation. Intra-molecular (Fig. 1B, Lat) and inter-molecular (Fig. 1B, Pol) loop insertion lead to the formation of latent and polymeric α_1_-antitrypsin, respectively. The β-hairpin model (Yamasaki et al., 2008) suggests no relationship between a native-latent pathway and polymerisation. Conversely, the C-terminal domain swap model (Yamasaki et al., 2011) suggests that the latent state may be an intermediate prior to polymer formation. Indeed, neuroserpin, another member of the serpin superfamily associated with encephalopathy and dementia, is able to readily polymerise from a latent conformation (Onda et al., 2005). The latent conformer has also been reported in other members of the serpin superfamily, including plasminogen activator inhibitor-1 (PAI-1) (Fjellstrom et al., 2013), antithrombin (Chang and Lomas, 1998, Corral et al., 2007, Laschke et al., 2004, Mushunje et al., 2004) and α_1_-antichymotrypsin (Gooptu et al., 2000). However, latency in α_1_-antitrypsin and its association with polymerisation remained unexplored in the absence of a suitable probe.

We report the development of a conformer-specific MAb (1C12) for latent α_1_-antitrypsin, and describe a robust methodology for using it to demonstrate and quantify levels of latent α_1_-antitrypsin in biological samples. Moreover, we have used the 1C12 MAb in combination with the polymer-specific 2C1 antibody to assess the association of the latent and polymeric conformers *in vitro*, in disease and during augmentation therapy. We have characterised the association of latency and polymerisation: (i) with *in vitro* kinetic studies using purified proteins; (ii) in a cell model of α_1_-antitrypsin deficiency; (iii) in biological samples of human liver; (iv) in human plasma and (v) in therapeutic preparations of α_1_-antitrypsin.

## 2 Materials and methods

### 2.1 Generation of monoclonal antibodies specific for the latent conformer of α_1_-antitrypsin

Purified latent α_1_-antitrypsin was prepared (Lomas et al., 1995) and used to immunise mice. Mouse spleen cells were isolated and hybridised with myeloma cells to generate hybridomas as described previously (Irving et al., 2011, Miranda et al., 2008). Iterative cloning and enzyme-linked immunosorbent assay (ELISA) were used to select hybridoma clones that produced antibodies that were specific for latent α_1_-antitrypsin. Several classes of antibodies with different binding specificities were identified, with the most interesting being the 1C12 that specifically recognised latent α_1_-antitrypsin. MAb 1F10 was identified that recognised both the latent and cleaved conformers of α_1_-antitrypsin. This antibody was used in combination with 1C12 in sandwich ELISA for quantifying the latent conformer (Supplementary Fig. 1).

### 2.2 Protein sample handling, gel electrophoresis and western blot

Plasma purified native *M* and *Z* α_1_-antitrypsin (Lomas et al., 1993) were stored in −80 °C and handled on ice for experiments. For ELISA and western blot analysis, cell and serum samples were also processed on ice. Non-denaturing (3–12% NativePAGE Bis–Tris gel) and SDS-PAGE (NuPAGE Bis–Tris, both from Life Technologies) gels were used to separate the samples. 6 M urea gel was prepared with 8% acrylamide (w/v) and 0.375 M Tris buffer. In western blot analysis, proteins were transferred onto nitrocellulose-membranes that were then blocked with buffer containing PBS, 0.05% Tween-20 (v/v), 5% skim-milk (w/v) and then incubated with the primary antibody in TBS (0.05% Tween-20 (v/v), 5% BSA (w/v)) overnight. The membranes were incubated with HRP-labelled secondary antibody for 1 h in blocking buffer and developed using enhanced chemiluminescence (Thermo Scientific, Rockford, IL USA)

### 2.3 MAb studies of polymer and latent formation *in vitro*

Plasma purified native *M* and *Z* α_1_-antitrypsin (Lomas et al., 1993) were incubated at 200 μg/ml in 20 mM Tris buffer, pH 7.4, with and without sodium citrate (0.7 M). *M* and *Z* α_1_-antitrypsin were heated at 48 °C and 40 °C, respectively. The choice of temperature was determined by temperature gradient analysis (Supplementary Fig. 3). Incubations were monitored by taking samples for analysis every 12 h (from 0 to 108 h, 10 data points). Samples were analysed by non-denaturing and 6 M urea PAGE, and quantified by sandwich ELISA using the 2C1 antibody for polymers (Ekeowa et al., 2010, Miranda et al., 2010) and the 1C12 antibody for latent α_1_-antitrypsin.

### 2.4 MAb detection of polymeric and latent α_1_-antitrypsin deficiency in a cell model

Chinese hamster ovary (CHO) cells were stably transfected to express *M* or *Z* α_1_-antitrypsin (Ordonez et al., 2013). The expression of α_1_-antitrypsin was induced by 1 μg/ml doxycycline (dox), and cells were cultured for 144 h (six days) before the detection of latent or polymeric α_1_-antitrypsin. The cells were also cultured with 0.5, 1, 2, 5, 10 or 20 μg/ml Brefeldin A, an inhibitor of endoplasmic reticulum (ER) to Golgi transport, for 30, 60, 90, 120 and 240 min. Furthermore, a range of compounds (in combination or separately) was used that interfere with different cellular pathways: tunicamycin (2.5 μg/ml), thapsigargin (250 nM) (each in the absence and presence of 5 μM lactacystin), and with 5 μM lactacystin alone. Tunicamycin and thapsigargin induce protein misfolding and ER stress, whereas lactacystin inhibits proteasome function. Media and/or cell lysates were collected from each condition, and analysed by ELISA for the presence or absence of polymeric and latent α_1_-antitrypsin.

### 2.5 Immunohistochemistry of human liver sections

Formalin-fixed, paraffin embedded human liver tissues were available from 30 individuals across a range of genotypes (Table 1) and two control samples. These were explants or biopsies from individuals undergoing investigation for α_1_-antitrypsin deficiency in a tertiary referral centre. Twenty-three individuals had cirrhosis (Ishak (Ishak et al., 1995, Standish et al., 2006) stage 6) and seven had minimal fibrosis (Ishak stage 1 or 2). Unless specified, all steps were performed at room temperature. The tissue slides were deparaffinised by serial incubation in xylene and decreasing concentration of ethanol. Antigen was retrieved by 10 mM sodium citrate buffer pH 6.0 at sub-boiling temperature for 10 min. For fluorescence microscopy, tissue slides were incubated overnight at 4 °C with two primary antibodies simultaneously, an in-house rabbit polyclonal antibody (2 μg/ml) that detects all conformers of α_1_-antitrypsin and the mouse MAb 1C12 (1:10 dilution of hybridoma cell culture medium) that is specific for latent α_1_-antitrypsin. This was followed by incubation with an anti-rabbit secondary antibody conjugated with tetramethyl rhodamine isothiocyanate (2 μg/ml), and an anti-mouse secondary antibody conjugated with fluorescein isothiocyanate (FITC, 2 μg/ml) for 1 h (both from Abcam, Cambridge, UK). A second liver tissue slide from the same individual was processed using the same procedure but stained with 2C1 instead of 1C12, for detection of polymeric α_1_-antitrypsin (Miranda et al., 2010). Tissue slides were viewed with a Carl Zeiss Laser Scanning Microscope 510 (Carl Zeiss Inc., Jena, Germany). For the bright-field microscopy, after deparaffinisation and antigen-retrieval, tissue slides were incubated overnight at 4 °C with one primary antibody, 1C12 or 2C1 on adjacent tissue slides from the same subject. Tissues were then processed with 0.3% (v/v) hydrogen peroxide, followed by incubation for 1 h with a secondary anti-mouse antibody labelled with horseradish peroxidase (HRP) and developed with diaminobenzidine (DAB, both from Sigma-Aldrich Co., Dorset, UK). The concentration of the antibodies used was the same as in the fluorescence microscopy. Tissue slides were counter-stained with Harris haematoxylin (Sigma-Aldrich Co., Dorset, UK) and viewed with a Zeiss Axioskope II (Carl Zeiss Inc., Jena, Germany) microscope. Polymer scores were determined using Image J (Schneider et al., 2012), by segmenting the polymer staining signals of the 2C1 antibody and measuring the fraction of the isolated area containing positive signals *versus* the total area analysed (embedded function of Image J, standard processing of immunohistochemistry staining). Regions for measurement were selected at random. Since samples contained little latent α_1_-antitrypsin, a binary system (+/−) was used to assess the presence or absence of this conformer.

**Table 1:** Assessment of fibrosis grade, polymer score and the presence of latent α_1_-antitrypsin in liver tissue from individuals with a range of α_1_-antitrypsin genotypes.

Sample No.	Genotype	Fibrosis grade	Polymer Score (%)	Latent (+/−)
1	MM	0	–	−
2	MM	0	–	−
3	MS	6	5	−
4	FZ	6	18	−
5	MZ	6	29	−
6	MZ	6	26	−
7	MZ	6	13	−
8	MZ	6	8	−
9	MZ	6	35	−
10	MZ	6	5	−
11	MZ	6	26	−
12	MZ	6	18	−
13	MZ	2	18	−
14	MZ	6	60	−
15	MZ	6	3	−
16	MZ	6	18	−
17	MZ	6	10	−
18	MZ	6	7	−
19	MZ	6	0.4	−
20	MZ	6	14	−
21	SZ	1	18	−
22	SZ	6	17	−
23	ZZ	6	69	+
24	ZZ	6	33	+
25	ZZ	6	59	+
26	ZZ	1	43	−
27	ZZ	1	11	−
28	ZZ	2	4	−
29	ZZ	1	3	−
30	ZZ	6	15	+
31	ZZ	6	14	+
32	ZZ	1	4	−

### 2.6 Immunoprecipitation and analysis of augmentation therapy

Immunoprecipitation (Tan et al., 2014) was carried out to confirm the ability of 1C12 to specifically detect latent α_1_-antitrypsin in plasma samples. Sepharose G beads were incubated with the 1C12 MAb and then with 1 μl of a plasma sample containing latent α_1_-antitrypsin as well as a plasma sample containing no latent α_1_-antitrypsin. A positive control of purified latent α_1_-antitrypsin, and three negative controls of immunoprecipitation with no added plasma, blotting of sepharose G beads and the 1C12 antibody were also included. The immunoprecipitated protein was eluted by dithiothreitol (DTT), and separated by SDS-PAGE followed by western blot analysis using the rabbit polyclonal antibody to detect α_1_-antitrypsin. A commercial preparation of augmentation therapy (Prolastin, Talecris Biotherapeutics Inc., North Carolina, US) was reconstituted according to manufacturer's instructions and diluted to 1 mg/ml stock for electrophoresis and western blot analysis.

### 2.7 ELISA screening of human plasma samples

811 Plasma samples (518 from the Alpha-1 Antitrypsin Genetic Modifier Study (Demeo et al., 2007) and 293 from the Alpha-1 Foundation DNA and Tissue Bank) (Tan et al., 2014) of mixed α_1_-antitrypsin genotypes (number): MM (200), MZ (20), ZZ (538), SZ (20), FZ (5), SS (3), MS (20), ZMheerlen (3) and ZMmalton (2), were analysed by ELISA (Cohort I). A further cohort of 116 plasma samples from the Alpha-1 Foundation DNA and Tissue Bank with information on augmentation therapy was also examined (Cohort II). The augmentation therapy used in Cohorts I and II was Prolastin. Furthermore, 24 and 33 samples from individuals receiving two other augmentation drugs, Aralast and Zemaira, were also analysed (Supplement Table 1). The screening analysis was composed of two steps: (i) a qualitative assay to determine the presence or absence of latent α_1_-antitrypsin; (ii) positive samples detected in (i) were further processed in a quantitative assay to determine the amount of latent α_1_-antitrypsin present. Briefly, in step (i) the plates were coated overnight with 2 μg/ml antigen purified rabbit polyclonal α_1_-antitrypsin antibody. Standard purified latent α_1_-antitrypsin (positive control) and subject samples were diluted in blocking buffer (1:200 for plasma samples) and incubated for 2 h. The wells were washed and incubated with the 1C12 MAb culture medium supernatant diluted in blocking buffer (1:50) for 2 h. Bound 1C12 antibody was detected with anti-mouse antibody labelled with HRP, and the reaction was developed with 3,3′,5,5′-tetramethylbenzidine (TMB) substrate solution (Sigma-Aldrich Co., Dorset, UK) and stopped with 1 M sulfuric acid. Absorption at 450 nm was measured using a plate reader (Molecular Devices, Thermo-max microplate reader). This assay distinguished between “latent-positive” and “latent-negative” samples, but its use in quantifying latent α_1_-antitrypsin was restricted by the limited linearity of the standard curve. Therefore, a quantitative assay with good linearity in regression analysis was developed using two monoclonal antibodies, 1F10 and 1C12 (Supplementary Fig. 1). Samples that gave a positive signal for latent α_1_-antitrypsin were analysed in step (ii) in which the plates were coated with 5 μg/ml 1F10 MAb that binds both latent and cleaved α_1_-antitrypsin. Serial dilutions of purified latent α_1_-antitrypsin (standard curve) and plasma samples diluted (1:2000) in blocking buffer, were incubated for 2 h, followed by detection using 0.5 μg/ml purified 1C12 antibody directly conjugated with HRP. The conjugation was carried out using EZ-link plus activated peroxidase kit (Thermo Fisher Scientific, Cramlington, UK). Reaction development and plate reading were the same as in step (i). The latent α_1_-antitrypsin concentrations were determined by regression analysis from standard curves.

### 2.8 Statistical analysis

The ELISA characterisation of monoclonal antibodies, *in vitro* kinetic dynamics of polymer and latent α_1_-antrypsin and the cell experiments were repeated three times. Data are presented with standard error of the mean (SEM). For the polymer scoring 20 images were analysed per biopsy sample, and the mean values of area fraction were recorded. The relation between levels of total and latent α_1_-antitrypsin was fitted using the linear function in EXCEL and the quality of fitting (R^2^, values close to 1 indicate good correlation) was calculated as the square of the Pearson product moment correlation coefficient.

### 2.9 Ethical approval

The study was approved by local Institutional Review Boards and all subjects provided informed consent.

## 3 Results and discussion

### 3.1 Development of conformer-specific monoclonal antibodies

**Fig. 2:**
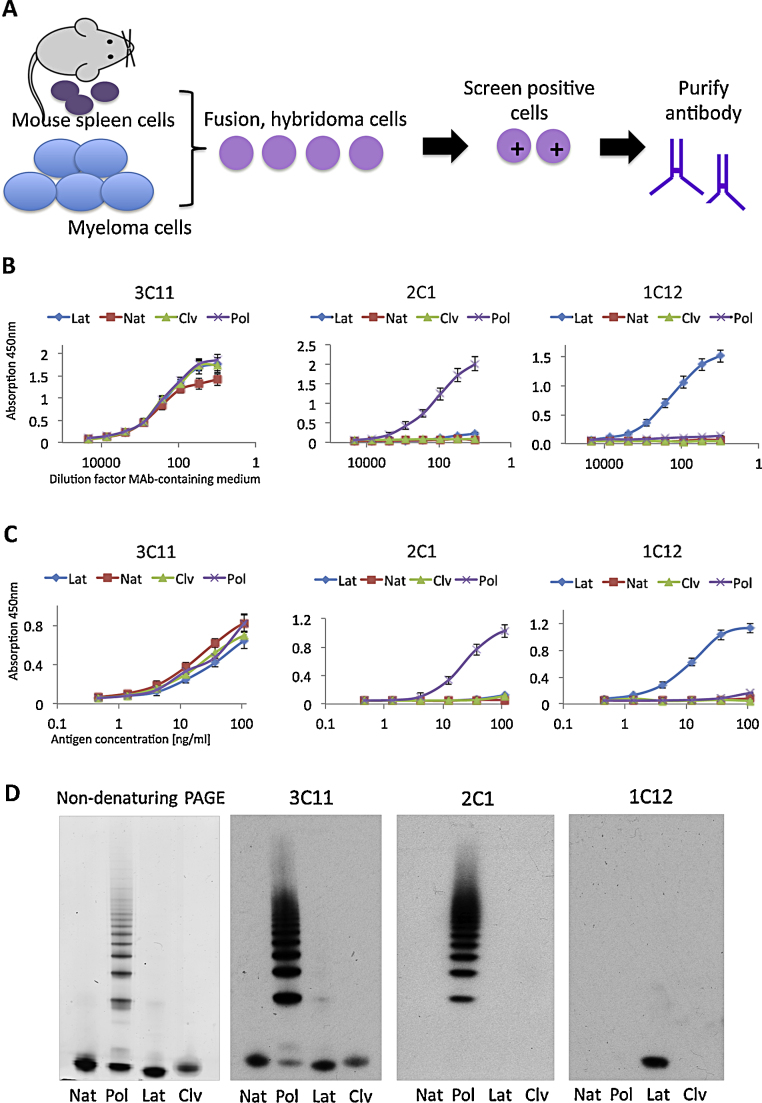
*Development of MAbs.* (A) Development procedure of MAbs. (B) Validation of MAbs specificity by antigen ELISA. The 3C11 and 2C1 MAbs were included as controls. The 3C11 MAb binds to all conformers of α_1_-antitrypsin whilst the 2C1 MAb recognises only the polymeric conformer as previously described (Miranda et al., 2010). The 1C12 MAb was identified that recognised only the latent conformer of α_1_-antitrypsin. (C) Validation of MAbs by sandwich ELISA. (D) Western blot analysis of non-denaturing gels with the 3C11, 2C1 and 1C12 MAbs respectively. The binding specificity of the MAbs to the protein conformers was consistent in (B)–(D). Results are *n* = 3 with SEM.

Immunisation of mice with purified latent α_1_-antitrypsin, and the screening of hybridoma cell lines derived from the mouse spleen cells, led to the identification of the 1C12 MAb that specifically recognised the latent conformer of α_1_-antrypsin (Fig. 2). In both antigen and sandwich ELISAs, the 1C12 MAb detected only the latent conformer and no other conformations including the native, cleaved and polymeric forms of α_1_-antitrypsin (Fig. 2B and C). Two control MAbs were included: 3C11 that detects all conformers of α_1_-antitrypsin and the previously characterised 2C1 antibody that detects only polymers (Miranda et al., 2010). MAb recognition of α_1_-antitrypsin conformers was also assessed by non-denaturing PAGE and western blot analysis (Fig. 2D), which yielded results that were consistent with the data from the ELISAs. Importantly, the binding specificity of the 1C12 MAb to the latent form of the *Z* mutant is the same as observed for the latent form of *M* α_1_-antitrypsin (Supplementary Fig. 2).

### 3.2 MAb studies of α_1_-antitrypsin latency and polymerisation *in vitro*

**Fig. 3:**
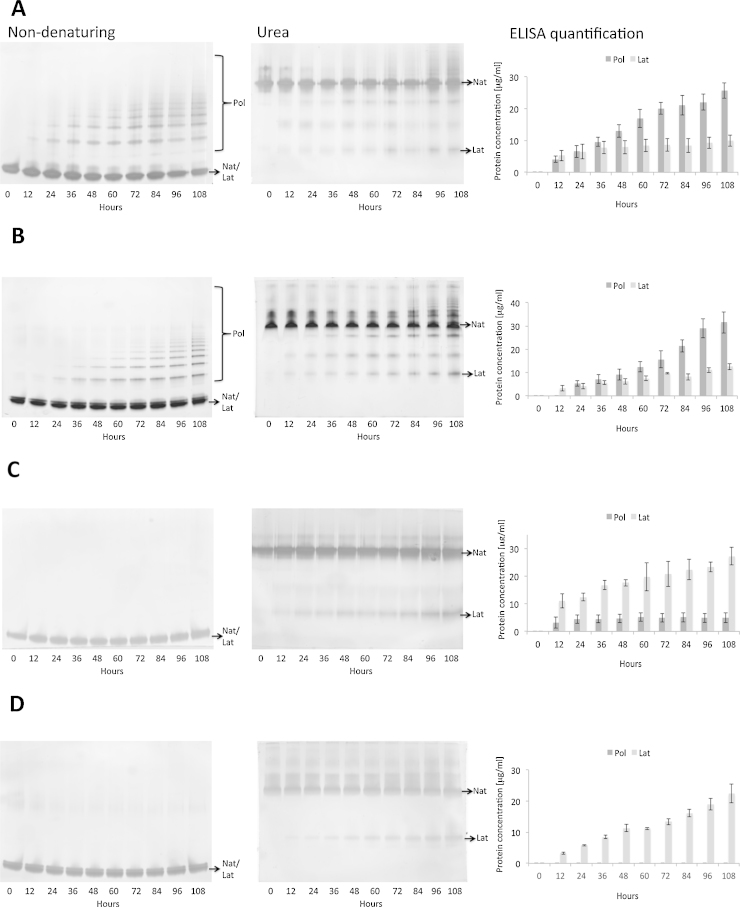
*MAb studies of α_1_-antitrypsin polymerisation and latency in vitro.* Native *M* and *Z* variants of α_1_-antitrypsin (200 μg/ml) were heated with or without sodium citrate at 48 and 40 °C, respectively. Samples were taken every 12 h for analysis from 0 to 108 h. (A) *M* α_1_-antitrypsin, without sodium citrate. (B) *Z* α_1_-antitrypsin, without sodium citrate. (C) *M* α_1_-antitrypsin, with sodium citrate. (D) *Z* α_1_-antitrypsin, with sodium citrate. Samples were analysed by non-denaturing PAGE (left) for visualisation of polymers, on a urea PAGE (middle) for separation of the latent conformer as the latent and the residual native conformers had a similar migration on a non-denaturing gel (left, Nat/Lat), and in sandwich ELISA (right) using the 2C1 MAb for detecting the polymer and 1C12 for the latent conformer. Results are *n* = 3 with SEM.

The availability of the conformer-specific antibodies allowed the investigation of the formation of α_1_-antitrypsin conformers *in vitro*. This was studied in a time course by incubating purified native monomeric *M* and *Z* α_1_-antitrypsin at equivalent polymerogenic temperatures (Supplementary Fig. 3) with and without sodium citrate. In the absence of sodium citrate, *M* and *Z* α_1_-antitrypsin formed both polymeric and latent conformers (Fig. 3A and B). Levels of the latent conformer were low and stabilised relatively early while polymer levels increased steadily over time. Sodium citrate stabilises the monomeric form of α_1_-antitrypsin and favours the pathway to latent α_1_-antitrypsin (Lomas et al., 1995). In the presence of sodium citrate, the polymerisation pathway was significantly inhibited, resulting in dominant production of the latent conformer for both *M* and *Z* α_1_-antitrypsin (Fig. 3C and D). The kinetics and relative quantities of the two conformers produced without manipulating the pathways (Fig. 3A and B) imply that the latent conformer is unlikely to be a direct precursor of polymers formed in these non-denaturing conditions, but rather a by-product of the polymerisation pathway.

### 3.3 Polymeric, but not latent, α_1_-antitrypsin was inducible in a cell model of α_1_-antitrypsin deficiency

**Fig. 4:**
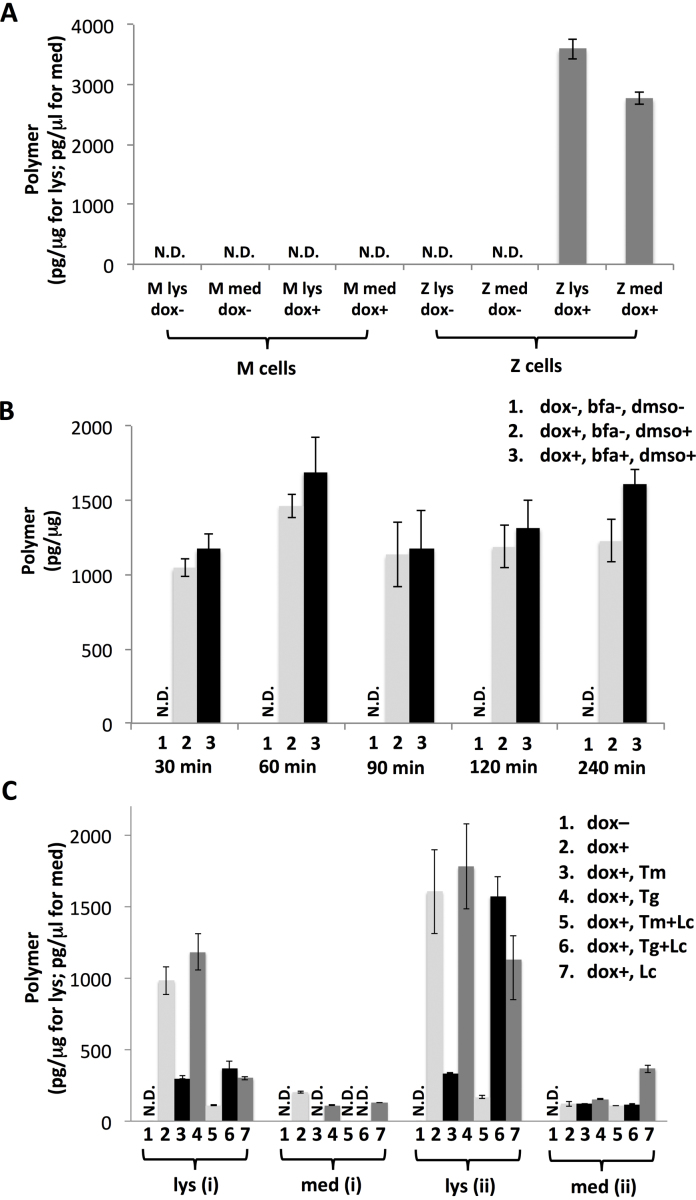
*Polymer was inducible in a cell model of Z α_1_-antitrypsin deficiency, whilst latent α_1_-antitrypsin was not detected.* CHO cells were stably transfected to express *M* (wild-type) or *Z* (mutant) α_1_-antitrypsin and expression was induced with doxycycline (dox) (Ordonez et al., 2013). ELISA assays were used to quantify the latent and polymeric α_1_-antitrypsin. Three different experiments were performed: (A) induction of *M* and *Z* α_1_-antitrypsin with dox. Cell culture media (med) and cell lysates (lys) were collected after 144 h (six days). There was no detectable latent α_1_-antitrypsin (data not shown), whilst abundant polymers were detected in the *Z* cells. (B) Cells were treated with brefeldin A (BFA, 5 μg/ml). BFA was dissolved in dmso and so a solvent control was included (column 2). No polymers or latent α_1_-antitrypsin were detected in cells expressing *M* α_1_-antitrypsin and no latent α_1_-antitrypsin was detectable in cells expressing *Z* α_1_-antitrypsin (data not shown). The figure shows polymer levels in the *Z* cell lysates. (C) CHO cells that express *Z* α_1_-antitrypsin were treated with combinations of tunicamycin (*T_m_*), thapsigargin (*T_g_*) and lactacystin (*L_c_*) as described in the methods section. Two protocols were used to administer the compounds: (i) cells were first treated with the compounds for 4 h, followed by the addition of dox and incubation for a further 12 h; (ii) cells were first treated with dox for 7 h followed by addition of the compounds and incubation of a further 12 h. These two protocols are denoted (i) and (ii). Media (med) and cell lysates (lys) were analysed for latent (negative, data not shown) and polymeric α_1_-antitrypsin. N.D.: not detected. Results are *n* = 3 with SEM.

We investigated the formation of polymeric and latent conformers in a cell model of α_1_-antitrypsin deficiency. CHO cells were stably transfected to produce comparable levels of total *M* and *Z* α_1_-antitrypsin (Ordonez et al., 2013). The cells were treated with a range of compounds in combination or separately (Fig. 4). CHO cells expressing *Z* α_1_-antitrypsin form intracellular polymers and provide a model system for studying ER overload and cellular pathways of polymerisation. Brefeldin A was used to block ER-Golgi transport to increase the amount of protein in the ER and so induce formation of latent α_1_-antitrypsin. Tunicamycin and thapsigargin were used to induce protein misfolding and ER stress to assess whether latent α_1_-antitrypsin might arise when chaperones were diverted to process misfolded proteins. Lactacystin was used to block the proteasome to assess whether latent α_1_-antitrypsin might arise when degradation was inhibited. However in no case was latent α_1_-antitrypsin detected in ELISA assays despite there being high concentrations of polymers (Fig. 4B and C). The presence of both polymeric and latent conformers *in vitro* and the absence of latent α_1_-antitrypsin in the cell model imply that cells may not produce latent α_1_-antitrypsin even when kinetic constraints are removed. This suggests that the native conformer is chaperoned or thermodynamically stabilised by some mechanism within the ER milieu. Alternatively the latent state may be produced at very low levels (compared to polymers) and efficiently cleared by a more specific cellular mechanism that was not inhibited in these experiments. Since the production of the latent α_1_-antitrypsin significantly increased when the polymerisation pathway was inhibited (sodium citrate, Fig. 3C and D) in the cell-free *in vitro* condition, cell permeating polymerisation blockers for drug design might by analogy increase intracellular latent α_1_-antitrypsin.

### 3.4 Distribution patterns of polymeric and latent α_1_-antitrypsin in liver tissue

**Fig. 5:**
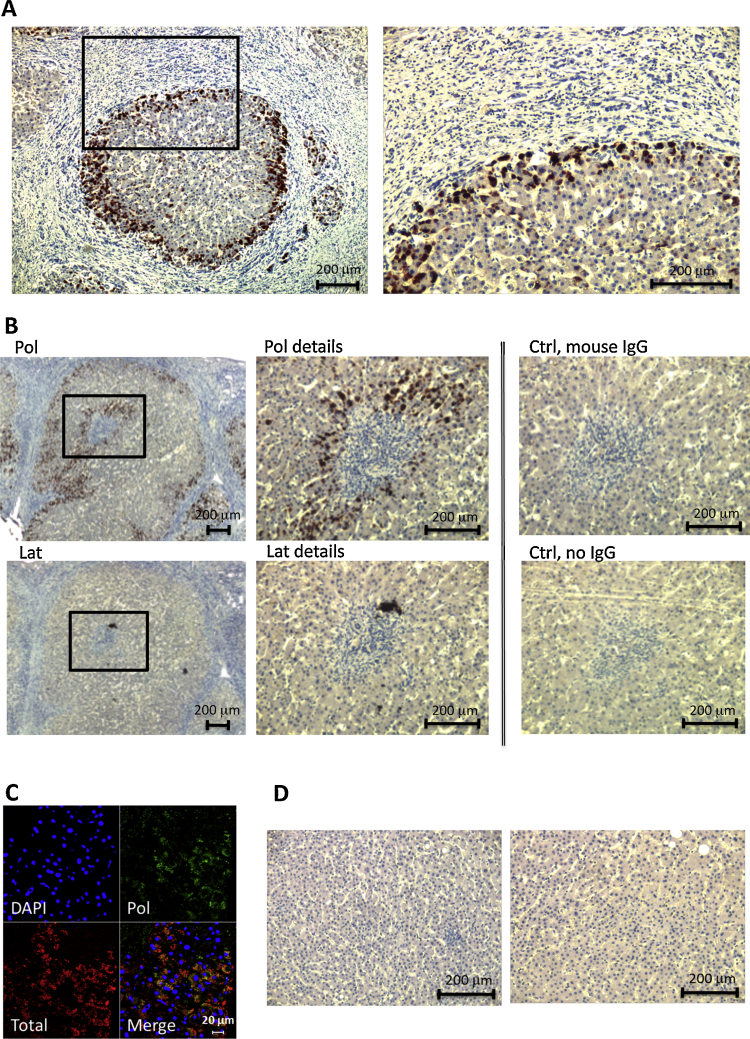
*Distribution patterns of polymeric and latent α_1_-antitrypsin in liver tissues.* Samples analysed in immunohistochemistry were from individuals who were not receiving augmentation therapy. (A) The typical location of polymers. Polymers (PiZZ) were most commonly observed at the periportal zone close to fibrous tissue (left, overview; right, magnified detail), suggesting a relationship between polymers and fibrosis. (B) Comparison of polymer and latent α_1_-antitrypsin staining. Serial slides (PiZZ) were stained using HRP–DAB, with the 2C1 (Pol) MAb, 1C12 (Lat) MAb, a non-specific generic mouse antibody (Ctrl, mouse IgG) and no primary antibody (Ctrl, no IgG). Images show an overview (left), magnified details of the positive signals (middle) and the control staining (right, separated by double lines). The presence of latent α_1_-antitrypsin in liver tissues was rare, whilst there were abundant hepatic polymers. (C) In addition to the HRP–DAB method, tissues were stained by the 2C1 MAb (and also 1C12 MAb, data not shown) and rabbit polyclonal antibody to co-localise the polymeric (Pol; green) and total α_1_-antitrypsin (Total; red), and examined by confocal microscopy. Exemplary image shows a moderate level of polymer staining in a PiZZ sample. (D) There is no staining in the control PiMM individual for polymers (left) or latent α_1_-antitrypsin (right). *n* = 30 individuals.

Liver tissue samples from 30 individuals with five different α_1_-antitrypsin genotypes (Table 1) and two PiMM control samples were analysed by immunohistochemistry and confocal microscopy. None of these individuals were receiving α_1_-antitrypsin augmentation therapy. The staining showed that polymers were most commonly located in the periportal zone in close proximity to portal tract fibrous tissue, forming ring-like patterns (Fig. 5A). High power images revealed that hepatocytes containing the greatest concentration of polymers were almost always in close proximity to fibroblasts. Whilst polymer was detected in tissue samples from all individuals who were homozygous or heterozygous for the *Z* allele (Table 1), only five individuals (all PiZZ) showed positive signals for latent α_1_-antitrypsin. The latent signals, if present, were only found in PiZZ individuals with advanced liver fibrosis. Comparing the signal intensity and distribution, the latent signals were much more sparse than the polymer signals, as observed in the HRP–DAB staining of serial tissue slides (Fig. 5B). In confocal microscopy, co-localisation of the conformer-specific (polymeric or latent) signal was assessed with the non-selective signal of total α_1_-antitrypsin stained by a rabbit polyclonal antibody (Fig. 5C).

The investigation of liver tissue samples showed there was abundant staining of polymers in all *Z* α_1_-antitrypsin homozygotes and heterozygotes, and there was also staining in an MS α_1_-antitrypsin heterozygote. The ability of the S variant to form polymers has previously been shown in secreted material purified *ex vivo* (Elliott et al., 1996). This is the first demonstration that S α_1_-antitrypsin polymers form within liver cells and that they are recognised by the 2C1 MAb. A polymer score was given to each sample (Table 1). Since little latent α_1_-antitrypsin was detected, the latent signal was not quantified but simply denoted as + or −. There was no more polymer staining in *Z* α_1_-antitrypsin homozygotes than heterozygotes. There was no longitudinal information available to correlate with accumulation of hepatic α_1_-antitrypsin polymers and the development of fibrosis. However, qualitative analysis showed a spatial association of polymers with fibrotic tissue (Fig. 5A). It may be that the local production of polymers by the nodules drives the proliferation of fibroblasts and the deposition of collagen. In comparison to polymers, little latent α_1_-antitrypsin was detected in liver tissues. Trace amounts of latent α_1_-antitrypsin were found in liver tissues of PiZZ individuals with advanced fibrosis. The observation from both the cell model and human liver samples implies that latent α_1_-antitrypsin does not accumulate without extreme stress. Alternatively it may be subject to a highly effective clearance mechanism that is only compromised in severe liver disease.

### 3.5 Circulating polymers arise endogenously, whilst plasma latent α_1_-antitrypsin originates from augmentation therapy

**Fig. 6:**
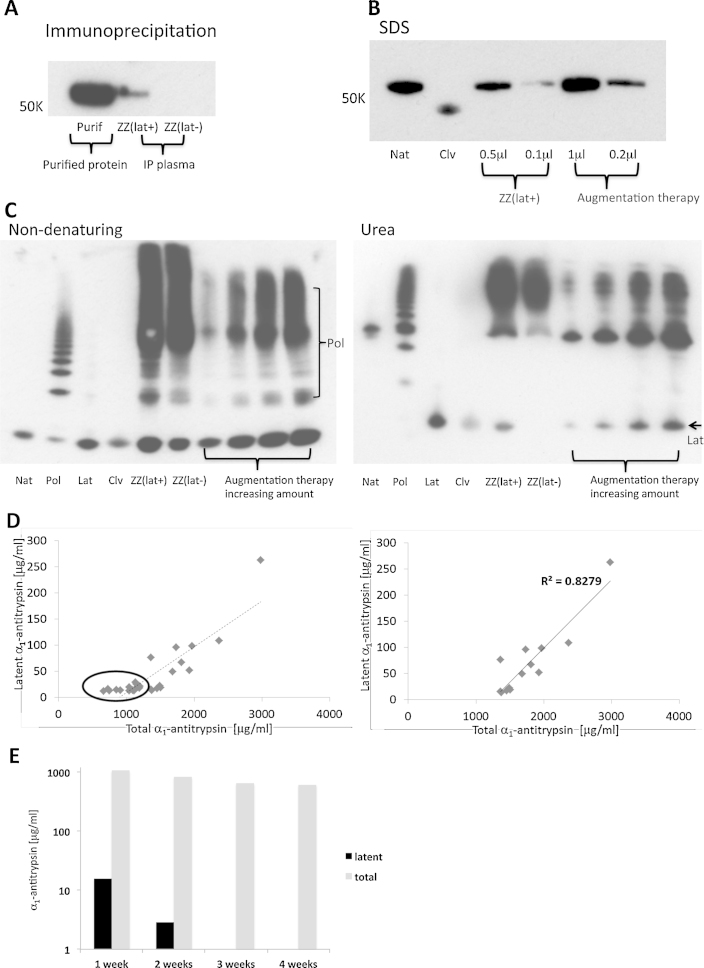
Plasma polymeric α_1_-antitrypsin arises endogenously, whilst plasma latent α_1_-antitrypsin originates from augmentation therapy. (A) The specificity of the 1C12 MAb was confirmed by using it to immunoprecipitate (IP) latent α_1_-antitrypsin from the plasma of PiZZ individuals with (ZZ(lat+), on augmentation) and without (ZZ(lat−), not on augmentation) a positive signal on ELISA. Proteins were transferred from the SDS gel to a membrane and probed with the rabbit polyclonal antibody. (B) The ZZlat(+) sample was analysed by western blot of an SDS PAGE together with the augmentation therapy. There was no detectable cleaved α_1_-antitrypsin in plasma or augmentation therapy. (C) The ZZ(lat+) and ZZ(lat−) samples, the augmentation therapy and purified native (Nat) and cleaved (Clv) controls were separated by non-denaturing and urea PAGE and subjected to western blot analysis for α_1_-antitrypsin using a rabbit polyclonal antibody. The western blot of the non-denaturing PAGE confirmed the presence of polymers in the ZZ(lat+) sample and in the augmentation therapy (Pol), which was consistent with our previous study (Tan et al., 2014). The western blot of the urea PAGE showed the presence of latent α_1_-antitrypsin in ZZ(lat+) plasma sample and in therapy (arrow, Lat). (D) Levels of the total and latent α_1_-antitrypsin are plotted for the latent-positive samples. At low α_1_-antitrypsin levels there is no association in the data points (left, circled). This may be due to the limited sensitivity of quantitative assays at lower protein abundance. At higher α_1_-antitrypsin levels, a linear correlation is found between latent and total α_1_-antitrypsin in the plasma of individuals receiving α_1_-antitrypsin augmentation therapy (right). (E) Levels of latent and total α_1_-antitrypsin at each time interval of therapy, averaged to the total number of samples assessed within the therapy group.

There is currently no cure for α_1_-antitrypsin deficiency. A treatment for the lack of circulating α_1_-antitrypsin is augmentation therapy, in which plasma-purified α_1_-antitrypsin is administered intravenously. We have analysed serum samples from individuals using Prolastin, Aralast and Zemaira. Prolastin is the most commonly prescribed augmentation, and was used by all individuals from Cohorts I (274 on therapy) and II (116 on therapy) on replacement therapy. In addition we analysed 24 and 33 serum samples from individuals on Aralast and Zemaira (Supplement Table 1). Due to the much smaller sample size and limited access to patient information for Aralast and Zemaira, a comparative analysis between the drugs is not possible. Thus, our substantive analysis focuses on the larger patient groups (Cohorts I and II) on Prolastin as these gave the requisite power for clear associations. We have previously shown that all individuals with PiZZ α_1_-antitrypsin deficiency have circulating polymers and that there is a weak correlation between the concentration of polymers and the presence of chronic obstructive pulmonary disease (Tan et al., 2014). We have also shown polymers were undetectable after 5 days (half-life 30 h), in serial plasma samples of a PiZZ individual who underwent liver transplantation, indicating that serum polymers arise from α_1_-antitrypsin produced within the liver. The 811 plasma samples from nine genotypes (Cohort I, Table 2) that were used in the previous study were re-screened for the presence of latent α_1_-antitrypsin with the 1C12 MAb. The specificity of 1C12 MAb was confirmed by using it to immunoprecipitate latent α_1_-antitrypsin from the plasma (Fig. 6A). The plasma screening using ELISA identified 63 PiZZ individuals with positive signals for latent α_1_-antitrypsin. 274 of the 538 PiZZ individuals were receiving α_1_-antitrypsin augmentation therapy. All the 63 latent α_1_-antitrypsin-positive individuals were receiving α_1_-antitrypsin augmentation, whereas the remaining 264 subjects who were not receiving augmentation therapy did not have any detectable latent α_1_-antitrypsin in their serum (*p* < 10^−14^, *χ*^2^ test). These findings suggest that the presence of circulating latent α_1_-antitrypsin was associated with the use of augmentation therapy. Further analysis using western blot of SDS, non-denaturing and urea PAGE (Fig. 6B and C) confirmed the presence of latent α_1_-antitrypsin in plasma samples and in the therapeutic formulation of α_1_-antitrypsin. Notably, augmentation therapy also contained polymers and the levels of polymers in individuals on therapy were generally higher than in those not on therapy (Tan et al., 2014). Our findings suggest that circulating polymer originates predominantly from endogenous material, whilst circulating latent α_1_-antitrypsin originates from augmentation therapy.

**Table 2:** Plasma samples in Cohort I and II.

Cohort I		
No. patients	Genotype	No. latent positive
538	ZZ	63
20	MZ	0
20	SZ	0
5	FZ	0
2	Zmmalton	0
3	Zmheerlen	0
3	SS	0
20	MS	0
200	MM	0
Cohort II		
No. patients	Genotype	No. latent positive
116	ZZ	25

Although 63 individuals showed a positive signal for latent α_1_-antitrypsin, the remaining subjects in the 274 individuals on augmentation therapy did not show detectable latent α_1_-antitrypsin. This may relate to the time that the protein was administered or the rate of clearance from the circulation. This information was not available in Cohort I and so a further cohort of 116 PiZZ subjects was investigated (Cohort II, Table 2, Table 3). In this cohort each blood sample was taken immediately prior to the administration of augmentation. The subjects were separated into four groups based on the frequency with which they received augmentation: every one, two, three or four weeks. Latent α_1_-antitrypsin was not detected in individuals receiving three or four weekly dosing; however, a proportion of individuals treated weekly or biweekly had a positive signal (Table 3). Although an accurate half-life for latent α_1_-antitrypsin cannot be determined without serial blood samples, we estimated the approximate half-life to be less than a week by comparing average levels in the blood samples. The concentration of circulating latent α_1_-antitrypsin correlated with the level of total α_1_-antitrypsin in individuals receiving augmentation (Fig. 6D). This further supports the observation that all circulating latent α_1_-antitrypsin is exogenous in origin.

**Table 3:** Latent and total α_1_-antitrypsin (AAT) levels for Cohort II.

Therapy frequency	Weekly	Fortnightly	3 weeks	4 weeks
Latent positive	21	4	0	0
Total tested	67	20	2	2
Mean latent for positive [μg/ml]	49.78	15.24	0.00	0.00
Mean total AAT for positive [μg/ml]	1501.06	1103.70	650.00	613.60
Mean latent for all [μg/ml]	15.65	2.90	0.00	0.00
Mean total AAT for all [μg/ml]	1058.01	829.28	650.00	613.60

The presence of polymeric and latent α_1_-antitrypsin in commercial preparation of augmentation therapy is consistent with the observation of polymeric and latent α_1_-antitrypsin being produced in cell-free conditions *in vitro* (Fig. 3). Inclusion of alternative, inactive conformers limits the quality and purity and hence functional activity of α_1_-antitrypsin augmentation therapy. The currently available MAbs and the methodologies developed can be used to detect and quantify the different inactive protein conformers in complex biological samples, and to improve the purity of α_1_-antitrypsin used in augmentation therapy. They will also be useful tools to study conformational transitions and to assess ‘on-’ and ‘off-pathway’ effects of novel polymerisation blocking therapies that are in development.

## 4 Concluding remarks

Both polymeric and latent α_1_-antitrypsin were detected during *in vitro* induction of polymerisation. In a cell model of α_1_-antitrypsin deficiency however, polymer but no latent α_1_-antitrypsin was detectable. In liver tissues, there was abundant polymer found in individuals with a range of α_1_-antitrypsin genotypes and liver fibrosis. In comparison, little latent α_1_-antitrypsin was detected in human liver tissues from PiZZ α_1_-antitrypsin individuals with advanced cirrhosis. Polymers were detected in plasma of all PiZZ individuals, whereas the latent α_1_-antitrypsin detected in circulation was shown to have arisen from the augmentation therapy. These data suggest that the latent conformer is a minor product of the polymerisation pathway *in vivo* and that the pathological polymer is unlikely to be assembled from latent-like protomers as has been proposed for the C-terminal domain swap model of polymerisation. Lastly, our findings have direct translational implications. Both latent and polymeric conformers of α_1_-antitrypsin are produced in industrial preparation of augmentation therapy, which compromises the purity and effectiveness of the drug. The ability to detect and quantify this using 2C1 and 1C12 MAbs will facilitate the optimisation of more functional drug preparations.

## Conflict of interest statement

The authors disclose no personal or financial conflicts of interest.
