# Supplementary material for: Characterising the association of latency with α1-antitrypsin polymerisation using a novel monoclonal antibody
Source: Int J Biochem Cell Biol. 2015 Jan;58:81–91. doi: 10.1016/j.biocel.2014.11.005 (PMC4305080; doi:10.1016/j.biocel.2014.11.005)
Supplement: Supplementary file 1 [file mmc1.docx]

SUPPLEMENTARY INFORMATION

Supplementary Figure 1. **Standard curves for the sandwich ELISA assays.**


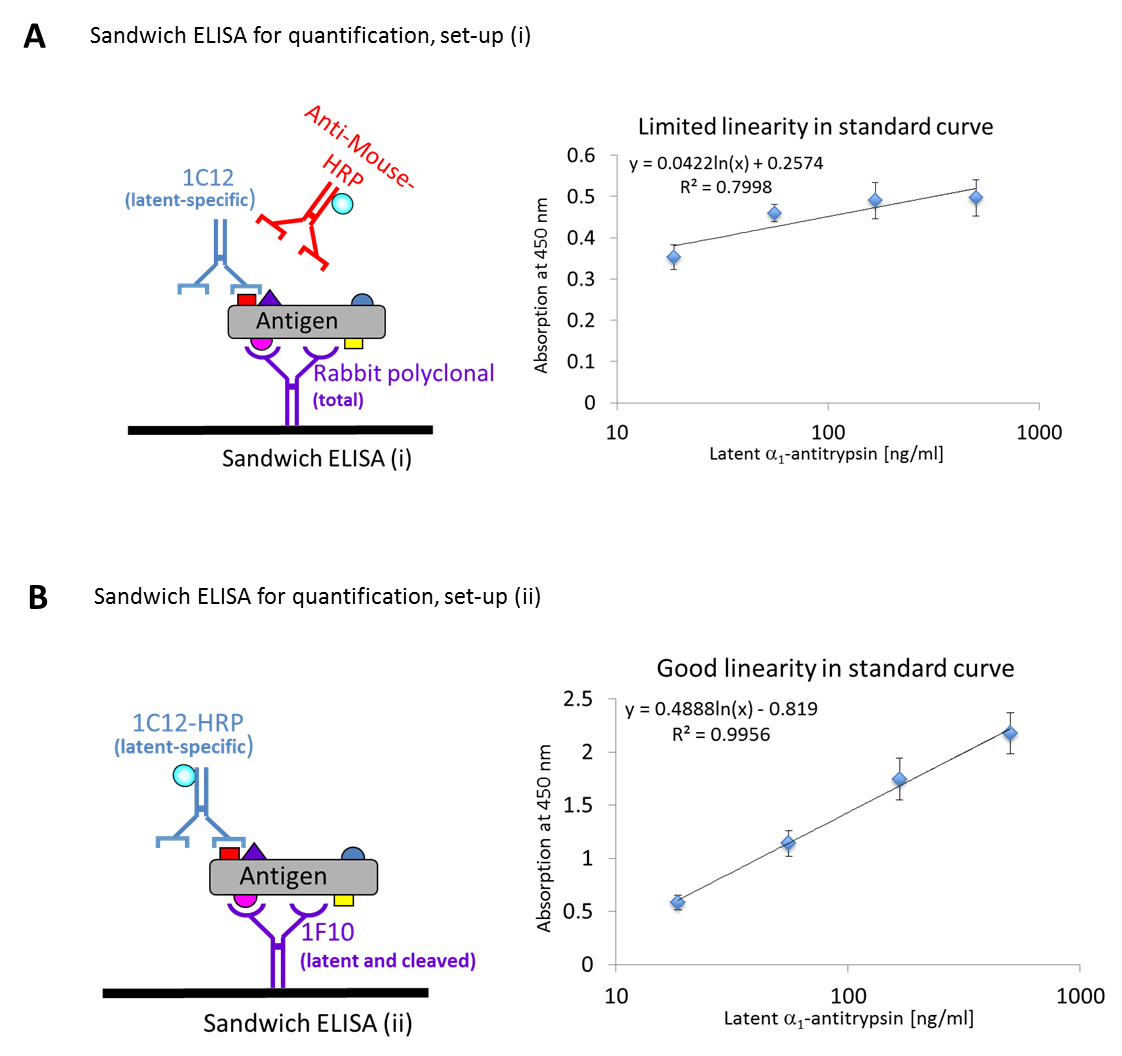


(A) Sandwich ELISA (i) used a rabbit polyclonal antibody to capture all conformers or total α_1_-antitrypsin. The detection monoclonal antibody 1C12 recognises only the latent conformation. An anti-mouse secondary antibody conjugated with HRP was used to detect 1C12. Standard protein (purified latent α_1_-antitrypsin) was used in serial dilutions to generate the standard curve (right). The linearity of the standard curve between 1000-10 ng/ml was not sufficient for quantitative analysis, although this assay was sensitive in excluding false-positives. Thus, another sandwich ELISA was developed as shown in (B). The 1F10 MAb that binds to latent and cleaved conformers of α_1_-antitrypsin was used as the capturing antibody. The 1C12-HRP conjugate that only binds to latent α_1_-antitrypsin was used as detection antibody. This greatly improved linearity of the standard curve made it suitable for quantifying the amount of latent α_1_-antitrypsin. The results are from 6 independent experiments.

Supplementary Figure 2. **The 1C12 MAb binds the latent conformer of both M and Z α_1_-antitrypsin.**


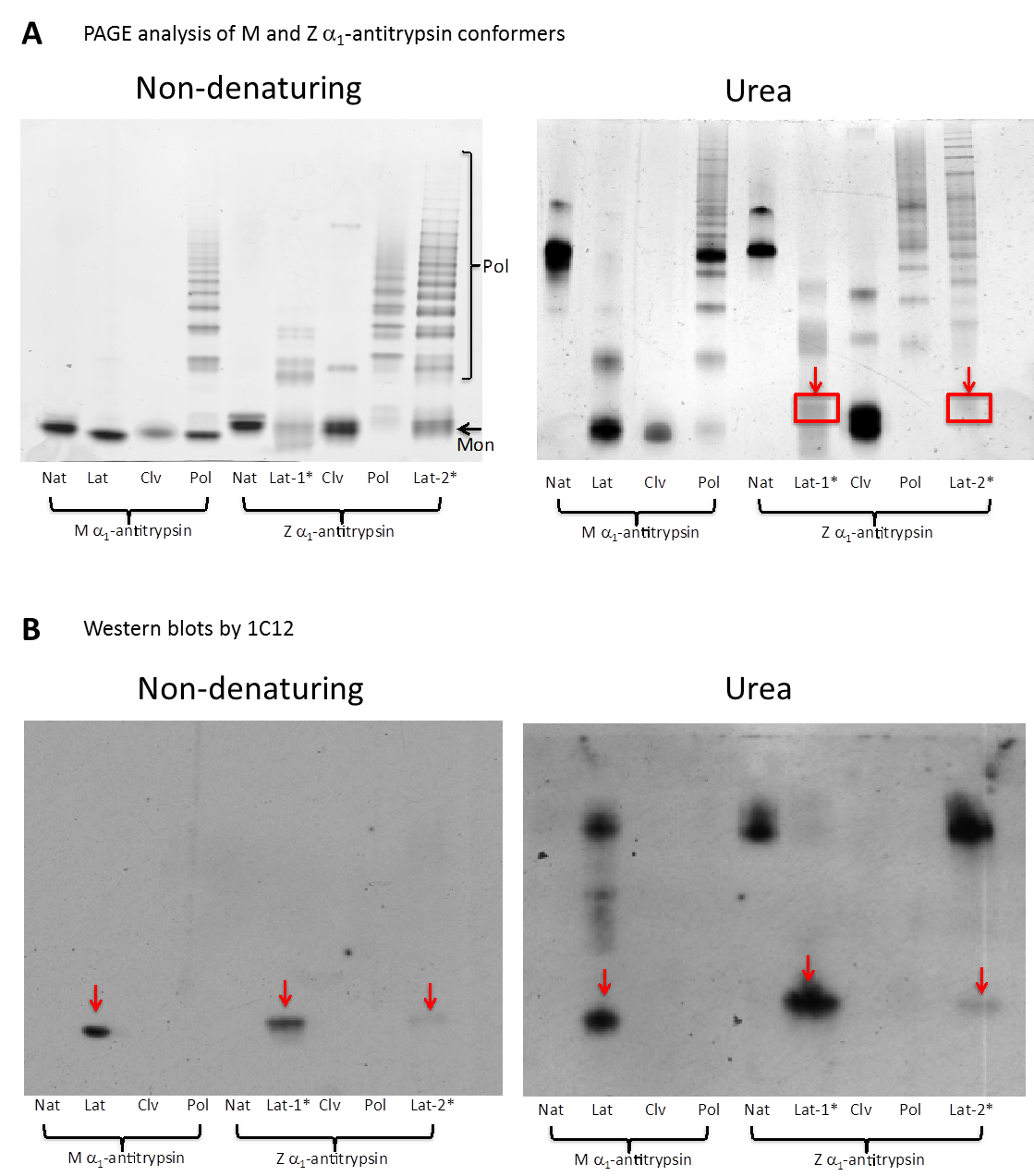


(A) Non-denaturing and urea PAGE analyses of different conformers of M and Z α_1_-antitrypsin. Native (Nat), latent (Lat), cleaved (Clv) and polymeric (Pol) M and Z α_1_-antitrypsin were analysed. All proteins were purified except for Lat-1* and Lat-2* in Z α_1_-antitrypsin, which were mixture of latent with other conformers. In non-denaturing PAGE, polymers appear as ladders (marked Pol) and Nat, Lat, and Clv migrate as monomers (Mon). In urea PAGE, the highly stable latent and cleaved conformers migrate towards the bottom of the gel. The Lat-1* and Lat-2* showed stable bands (red boxes, arrows), mixed with other conformers. (B) Western blot of the non-denaturing and urea PAGE with MAb 1C12. The 1C12 MAb detected the latent form in M α_1_-antitrypsin, and the latent bands in the mixture of conformers of Z α_1_-antitrypsin separated by non-denaturing and urea PAGE (red arrows). The additional bands detected on urea blot were likely artefacts caused by the denaturing condition of the urea gel blotting that interfered with antibody binding.

Supplementary Figure 3. The effect of temperature on the Z and M α1-antitrypsin.


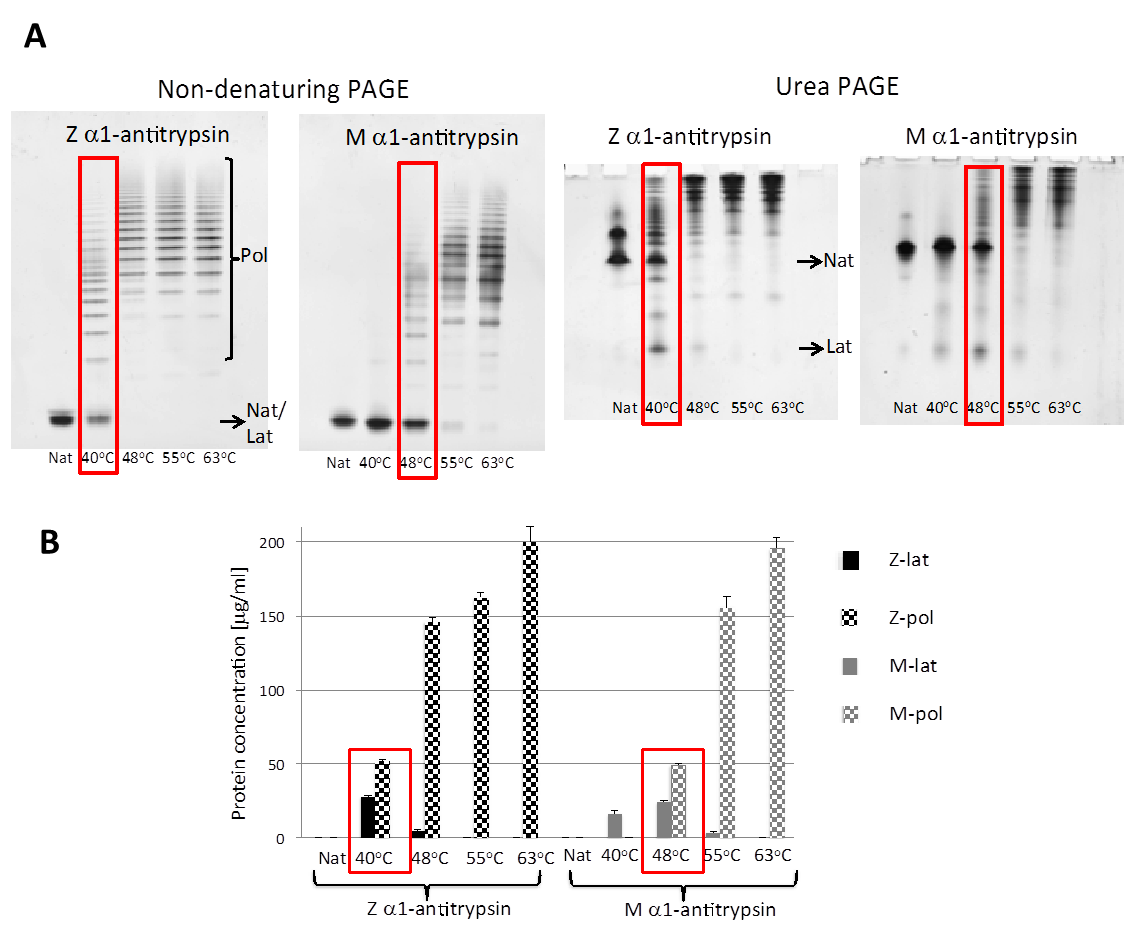


The temperature gradient analysis was designed to determine quasi-equivalent temperatures for the M and Z variants, because their thermodynamics profile at a given temperature is different {Lomas, 1993 #2;Dafforn, 1999 #3}. The M variant is more stable than the Z mutant. The melting temperature (T_m_) is 53.4 ^o^C for Z α_1_-antitrypsin but is 8 ^o^C higher (61.4 ^o^C) for M α_1_-antitrypsin {Lomas, 1993 #2;Dafforn, 1999 #3}. Therefore, we heated both variants (200 μg/ml) for 144 hours at four different temperatures: 40, 48, 55 and 63 ^o^C, with an increment of 7-8 ^o^C to match the difference in their melting temperatures. An unheated native protein control (Nat) was included in all analyses. (A) The resulting samples were analysed by non-denaturing PAGE for visualisation of polymer, and on urea PAGE to identify latent α_1_-antitrypsin. The non-denaturing PAGE showed that the conversion of native protein to polymer increased with increasing temperature for both M and Z α_1_-antitrypsin, with the Z protein starting to polymerise at 40 ^o^C where the M variant was still stable. Monomeric Z α_1_-antitrypsin was depleted after 144 hours at 48, 55 and 63 ^o^C, whereas the native M protein was depleted at 63 ^o^C and not at lower temperatures. The urea PAGE showed the formation of latent conformer was favoured at lower temperatures, and polymer at higher temperatures. The Z variant formed the latent conformer at 40 and 48 ^o^C, whilst the latent conformer could be detected in the M variant at 40, 48 and 55 ^o^C. Comparing these results, the most similar thermodynamic profile for M and Z variants was at 48 ^o^C for M and 40 ^o^C for Z α_1_-antitrypsin (highlighted in red boxes), respectively. (B) The same samples were also quantified in ELISA by using the 2C1 MAb for detection of polymers and 1C12 for the latent conformer. The ELISA result was in agreement with the PAGE analysis. Full conversion of native proteins to polymers were observed at 63 ^o^C for both M and Z variants, whereas fewer polymers were formed at lower temperatures at which the latent conformer was detectable. The highlighted data (red boxes) showed the temperature conditions at which similar patterns were observed for M and Z α_1_-antitrypsin. Therefore, in the time-course analysis we chose 40 and 48 ^o^C for the analysis of Z and M α_1_-antitrypsin respectively.

Table 1. Data of serum samples from individuals on augmentation drugs Aralast and Zemaira

| **Aralast** | | | |
| --- | --- | --- | --- |
| ID | Genotype | Liver Txp | Lat |
| 1 | ZZ | No | - |
| 2 | ZZ | No | - |
| 3 | ZZ | No | - |
| 4 | ZZ | No | - |
| 5 | ZZ | No | - |
| 6 | ZZ | No | - |
| 7 | ZZ | No | - |
| 8 | ZZ | No | - |
| 9 | ZZ | No | - |
| 10 | ZZ | No | - |
| 11 | ZZ | No | - |
| 12 | ZZ | No | - |
| 13 | ZZ | No | - |
| 14 | ZZ | No | - |
| 15 | ZZ | No | - |
| 16 | ZZ | No | - |
| 17 | ZZ | No | - |
| 18 | ZZ | No | - |
| 19 | ZZ | No | - |
| 20 | ZZ | No | - |
| 21 | ZZ | No | - |
| 22 | ZZ | No | - |
| 23 | ZZ | No | - |
| 24 | ZZ | No | - |
| **Zemaira** | | | |
| ID | Genotype | Liver Txp | Lat |
| 25 | ZZ | UnkNown | - |
| 26 | ZZ | No | + |
| 27 | ZZ | No | - |
| 28 | ZZ | No | - |
| 29 | ZZ | No | - |
| 30 | ZZ | No | - |
| 31 | ZZ | No | - |
| 32 | ZZ | No | - |
| 33 | ZZ | No | - |
| 34 | ZZ | No | - |
| 35 | ZZ | No | - |
| 36 | ZZ | No | + |
| 37 | ZZ | No | - |
| 38 | ZZ | No | - |
| 39 | ZZ | No | - |
| 40 | ZZ | No | - |
| 41 | ZZ | No | - |
| 42 | ZZ | No | - |
| 43 | ZZ | No | - |
| 44 | ZZ | No | - |
| 45 | ZZ | No | - |
| 46 | ZZ | No | - |
| 47 | ZZ | No | - |
| 48 | ZZ | No | - |
| 49 | ZZ | No | - |
| 50 | ZZ | No | - |
| 51 | ZZ | No | - |
| 52 | ZZ | No | - |
| 53 | ZZ | No | - |
| 54 | ZZ | No | - |
| 55 | ZZ | No | + |
| 56 | ZZ | No | - |
| 57 | ZZ | No | - |
|  |  |  |  |

No latent positive samples were found in Aralast users; three samples just above the detection limit of ELISA were found Zemaira users.
